# Optimizing hybrid energy systems for remote communities in Asia’s least developed countries

**DOI:** 10.1016/j.heliyon.2024.e29369

**Published:** 2024-04-17

**Authors:** Mengly Prum, Hui Hwang Goh, Dongdong Zhang, Wei Dai, Tonni Agustiono Kurniawan, Kai Chen Goh

**Affiliations:** a School of Electrical Engineering, Guangxi University, No.100 Daxue Road, Nanning, Guangxi, 530004, PR China; b College of the Environment and Ecology, Xiamen University, Fujian, 36110, PR China; c Department of Construction Management, Faculty of Construction Management and Business, University Tun Hussein Onn Malaysia, 86400, Parit Raja, Johor, Malaysia

**Keywords:** Rural electrification, Least developed countries (LDCs), Sustainability, Hybrid energy system (HES), Multi-criteria decision-making (MCDM)`

## Abstract

In least-developed countries (LDCs), electricity shortages are the primary barrier to economic and social growth. Some remote areas in LDC rely on diesel-based systems. However, renewable energy must be taken into account for generating electricity because of the uncertainty of diesel fuel prices and the emissions of carbon dioxide. Hybrid energy systems (HES) are becoming increasingly popular, which is unsurprising given the rapid advancement of renewable energy technologies, which have made them the preferred method to respond to the current unreliable electricity supply, reduce the impact of global warming that occurs from electricity production, and contribute to cost reduction. This study explores the feasibility of utilizing a combination of solar PV, wind energy, and battery systems with the existing diesel generator in four different locations in Cambodia, Laos, Myanmar, and Bangladesh. Hybrid optimization multiples for electric renewables (HOMER) is used as a tool for techno-economic analysis and finding the possible combination of solar PV, wind, diesel, and battery. The multi-criteria decision-making (MCDM) technique was used to verify all configurations obtained from HOMER’s results. This approach considers environmental, economic, and technological factors by utilizing the AHP, TOPSIS, EDAS, and PROMETHEEE II techniques. The results show that PV/diesel with batteries is the optimum solution. This hybrid system comprises 89% PV penetration, a cost of electricity (COE) of 0.257 $/kWh, an initial capital cost (IC) of $244,277, and a net present cost (NPC) of $476,216 for a case study in Cambodia. Furthermore, this system can reduce almost 51,005 kg/year of carbon dioxide compared to a diesel-only system, while the cost of electricity is reduced.

## Graphical abstract

To create your abstract, type over the instructions in the template box below.Fonts or abstract dimensions should not be changed or altered.

**Figure:**
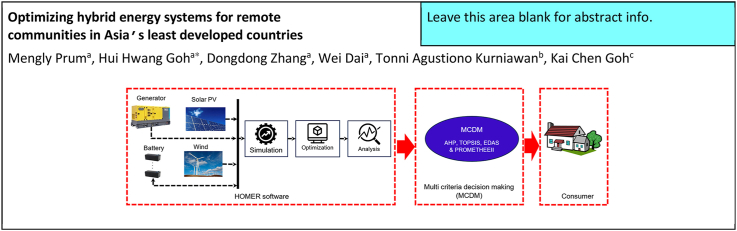

## 1 Introduction

The electricity shortage is the primary barrier to economic and social growth. Currently, more than 80 percent of the approximately 759 million individuals worldwide without access to electricity reside in rural areas [1]. Access to reliable and affordable electrical power is essential for accelerating the development of remote communities where grid connection is impractical, particularly in remote regions [2]. Certainly, remote regions in least-developed countries (LDCs) in Asia, including Cambodia, Laos, Myanmar, and Bangladesh, are not supplied with reliable and clean electrical energy due to a lack of power supply, and distribution lines cannot be extended to these areas due to low load demand and high investment costs. Furthermore, the countries of Cambodia, Laos, Myanmar, and Bangladesh primarily rely on traditional fossil fuels, including coal, natural gas, diesel oil fuel, and fuel oil, for the purpose of electricity generation. As a result, conventional approaches, such as employing a diesel generator, were the sole alternative for supplying electricity to isolated regions. Most of those remote areas rely on diesel-based systems. However, the primary drawbacks of these sources are their rapid rate of depletion and the negative environmental effects caused by the combustion process, and the electricity cost is also high [3].

Renewable energy (solar photovoltaic, wind, hydro, biomass, etc.) technology is one of the most significant strategies to fulfil the growing energy demand and reduce greenhouse gas emissions [4,5]. In Cambodia, the average sun shines for 8 h per day over the year. The amount of solar energy received per square meter in Cambodia is measured as recorded solar irradiation, and it is very powerful, with daily averages of 5 kWh/m^2^ and a peak of 5.6 kWh/m^2^ in the central region of the country [6]. In Laos, the potential for solar energy is estimated to be 4.4 kWh/square meter per day, within a range of 3.6–5.5 kWh/square meter per day and equating to 1800–2000 h of sunlight per year [7]. In Myanmar, the amount of solar energy received per square meter in Cambodia is measured as recorded solar irradiation, and it is very powerful, with daily averages of 5 kWh/m^2^. Bangladesh has abundant solar energy resources; 94% of the country's land receives 4–6.5 kWh/m^2^ of solar radiation, or 6.5 h of effective sunlight per day on average [8].

In order to respond to current electricity consumption and contribute to reducing the impact of global warming that occurs from electricity production and contributing to cost reduction, the integration of solar energy is an important part of solving the above intelligence effectively and efficiently. The exploration of new technology to increase renewable energy production is essential to getting more benefit. Furthermore, hybrid renewable energy systems are gaining popularity, which is not unusual given the rapid development of renewable energy technologies, which have made them the preferred method for powering electricity in remote areas. Nevertheless, the unreliability of renewable energy sources for a consistent energy supply might be attributed to their variable, seasonal, and ambiguous characteristics [[9], [10], [11]]. To meet the requirements of demand consumption, these systems must utilize either significant sustainable energy storage or a combination of existing primary generators. Hybrid energy systems, which consist of a combination of diesel generators and battery banks, have been proposed as a potential approach to mitigate dependence on battery storage and improve power supply reliability [[12], [13], [14]].

Multiple research projects have provided evidence that the optimization of hybrid energy system (HES) yields greater efficiency and cost-effectiveness. The research study conducted by Shoeb and Shafiullah et al. [15] evaluated the viability of utilizing a possible combination of photovoltaic (PV), diesel engines, and battery systems to supply electricity for agricultural purposes and meet the energy needs of rural regions in Bangladesh. In their study, Lao et al. [16] conducted an optimization analysis on several configurations of PV/Battery/Diesel systems in a rural district in Cambodia, with a focus on both technological and economic considerations. The PV/Diesel/Battery configurations assessed by Halabi et al. [17] were compared to various configurations, and it was found that the analyzed configurations offer the most favorable technological, economic, and environmental limitations. Guangqian et al. [18] employed hybrid harmonies searches simulated annealing (HS-SA) strategies to analyze the off-grid mixing of PV/Wind/Biodiesel/Battery systems with the goal of reducing life cycle costs (LCC). This present research study ascertained that the levelized cost of electricity for the PV/Biodiesel/Battery and Wind/Biodiesel/Battery systems amounted to $21,266 and $29,153, respectively. Ahmed et al. [19] analyzed an optimization study to enhance the technological and economical performance of a hybrid renewable energy system (HRES) containing PV, wind, biomass, and battery components that is connected to the grid. Consequently, the ideal configuration was found to be the HES, which exhibited net present expenses amounting to $180.2 million and energy costs of 0.057 $/kWh.

Furthermore, a considerable number of scholarly articles have examined the technological, economical, and environmental viability of different off/on-grid HES for the purpose of electrifying remote regions. In their study, Aziz et al. [20] conducted an optimization of PV/Hydro/Diesel/Batterie systems with the goal of satisfying the consumption requirements of rural populations in Iraq, including both technological and economic conditions. The outcome of the study indicates that the hydro/PV/diesel/battery approach emerges as the optimal economically efficient alternative, exhibiting an NPC of $113,201 and achieving a renewable energy percentage of 91.03%. In their study [21], the authors employed multiple-objective Crows search algorithms to optimize PV, diesel, and fuel cell systems. The main aim of their investigation was to evaluate the NPC and the probabilities of power supply losses (LPSP) in order to achieve their desired outcomes. This approach was utilized to guide their goal-oriented actions. The results indicated that the NPC amounted to $1.3 million in the absence of LPSP, which subsequently fell to $1.076 thousand with the implementation of a 10% LPSP. In order to address the varied consumption needs within Jubail Commercial City, Saudi Arabia, a comprehensive assessment of the technological and financial aspects of PV, wind, battery, and diesel systems was undertaken, drawing on the findings presented in Ref. [22]. Based on their analysis, it has been observed that an increase in electrical demand leads to a decrease in the electricity costs associated with PV/Wind/Battery/Diesel systems. Specifically, when the demand consumption is reduced from 11,160 to 3288 kWh per day, the electricity price rises from $0.18 to $0.24 per kilowatt-hour, while the electricity cost for Wind/PV systems amounts to $0.25 per kilowatt-hour. The study implemented by Odou et al. [23] employed the HOMER tools to optimize the viability of conducting the HRES in order to fulfill power supply to remote areas in Benin. The outcome of the study found that the integration of PV systems, batteries, and diesel generators emerged as the most economically efficient alternative. In their study, Mohamed et al. [24] employed particles swarms optimization (PSO) techniques to optimize a PV/Wind systems operating on a grid. Their findings indicated that the suggested approach of integrating PV/Wind with grid connectivity exhibited superior performance compared to other schemes. In their study, the authors in Ref. [25], employed mixed integer linear programming (MILP) to optimize a grid-integrated photovoltaic (PV) system. They assert that this optimization approach led to a significant energy savings of 64.16 percent. The authors employed PSO in their study, documented in Ref. [26], to optimize the solution involving the PV, battery, and diesel systems. They conducted a comparison of their PSO-based approach with the HOMER program. According to the findings of the inquiry, it was determined that the cost of COE is somewhat lower than that of HOMER, with corresponding values of 0.38 and 0.40 $/kWh. Moreover, photovoltaics made a comparable contribution to total energy production. In their study, Tan and Bu-Kar [12] examined the maximal dimensions and energy managements approach for a self-sufficient system containing a combination of PV, wind, and fuel cell technologies. The function of the EMS in determining system size is evidently crucial, with economic, technological, and environmental issues emerging as the foremost considerations in the optimization of HES sizing.

The researchers in this work employed the HOMER program to calculate the size of the HES components. The option of using HOMER was based on its user-friendly interface [27] and efficient search capabilities [28]. The analysis was enhanced by a thorough examination of the objectives function and the ensuing indicators of achievement, as well as using the usual HOMER software tool. Furthermore, the research presents a comprehensive framework for the implementation of Health Information Exchange Systems (HES), encompassing both the obstacles and potential advantages associated with their adoption.

The optimization strategies employed in several studies incorporate considerations of net present costs and energy expenses. However, the optimum technique does not take into account other economic, ecologically favorable, or other aspects. Therefore, the utilization of multi-criteria decision-making (MCDM) methods is becoming common in the process of choosing energy-efficient alternatives. The process of MCDM involves doing a set of assessments on a restricted set of options using a preset set of criteria [29]. The evaluation of HRES using the MCDM technique has been extensively examined in several research projects. The ideal option among thirteen choices was established by the author of [30] by the utilization of a mix of Analytic Hierarchy Process (AHP) and VlseKriterijumska Optimizacija I Kompromisno Resenje (VIKOR) methodologies. Prior to assigning weights to the criterion, experts in academia were consulted. Ultimately, it has been ascertained that compact, decentralized generation is the optimal choice for providing power to rural regions. A study performed by Solangi et al. [31] in Pakistan explored several renewable energy sources. The present study employed the AHP, Delphi, and Fuzzy-TOPSIS methodologies for the purpose of analysis. The Delphi approach was employed to identify the best critical criteria within the initial stratum. The Fuzzy-TOPSIS strategy was utilized to rank the resources subsequent to the utilization of the AHP method for evaluating the second stratum of criteria. The findings suggest that wind power emerged as the most advantageous energy source for fulfilling consumption demands, with hydropower, solar energy, and other sources following suit. In this investigation [32], the analysis of a HRES for the electrifications of a remote communities hamlet in Iran's northwestern area was conducted. The HOMER program was utilized for this purpose. Subsequently, the TOPSIS approach was employed to evaluate the system's performance in both off/on-grid scenarios, while considering environmental and financial factors. This analysis aimed to assess the feasibility and effectiveness of implementing the HRES in the given context. The ideal solution for an off-grid model was determined to be a blend of PV/Biogas/battery, while for an on-grid model, the optimal solution was found to be a mix of PV/Wind. In this study, in Ref. [33] established a two-stages MCDM model with the objective of identifying the best electrification approach structures in Egypt. This study employed the HOMER software to examine eleven potential scenarios, followed by the utilization of VIKOR, CODAS, and WASPAS for post-optimality analysis to ascertain the best possible design. The findings of this study indicate that the integration of photovoltaic (PV), wind, and battery technologies offers the greatest environmental advantages when compared to relying solely on diesel power or expanding the grid infrastructure. In their study, the authors introduced a novel mixed-MCDM approach to assess the best selection of HRES to fulfill the electricity consumption of a governmental organization in Iran [34]. Initially, the HOMER software tool was employed to investigate a feasibility evaluation of many options. Subsequently, the BWM, AHP, and FUCOM approaches were employed to prioritize weights according to the criteria. Lastly, TOPSIS and EDAS techniques were employed to rank all the scenarios. The findings confirmed that the grid, WT, and BES emerged as the most favorable alternatives in the challenging economic conditions.

Despite the fact that numerous studies have been conducted for a particular condition location, some studies' optimizations utilize net present costs and energy costs; other environmental, economic, and other factors are not considered during the optimization process. This study used HOMER software to investigate the feasibility of utilizing a combination of solar PV, wind energy, and battery systems with the existing diesel generator to respond to the current unreliable electricity supply, reduce the impact of global warming that occurs from electricity production, and contribute to cost reduction. Additionally, the present study proposed a novel approach that included the AHP, TOPSIS, EDAS, and PROMETHEE II methodologies used to verify all configurations obtained from HOMER’s results. This proposed methodology was utilized to identify the best suitable configuration for the HES, taking into account various elements such as environmental impact, economic viability, and technological feasibility. The primary sources of energy generation in Cambodia, Laos, Myanmar, and Bangladesh are fossil fuels, including coal, natural gas, diesel fuel, and fuel oil. Reliable electricity supply is critical for promoting socioeconomic growth and guaranteeing long-term sustainability within the framework of a country's energy security. Thus, the integration of renewable energy sources in the energy mix is critical to supporting the supply of energy and promoting sustainable growth with low environmental effects, which is in accordance with the SDG7 principles of Sustainable Development Goal 7 (SDG7) for the year 2030. SDG7 refers to the goal of increasing the number of sustainable, clean energy sources within the worldwide energy portfolio [1]. The subsequent findings represent the most noteworthy contributions to this inquiry.

• This study analyses the viability of employing a hybrid energy system (HES) composed of PV, wind, and backup battery technologies with the existing fossil fuel resources in terms of technical and economic aspects in order to solve the current unreliable electricity supply, reduce the impact of global warming and greenhouse gas emissions that occur from electricity production, and contribute to cost reduction.
• In this study, we compare the different system configurations, especially with the existing diesel-only systems, and propose the optimum sizing of various HES components.
• This study applies the decision-making method, including AHP, TOPSIS, EDAS, and PROMETHEE II techniques, to verify the results obtained from the HOMER results. It considers multiple factors, including environmental, economic, and technical considerations.

The subsequent illustration displays the comprehensive framework of the research. The second half of the paper provides a detailed account of the proposed system and its modeling. The subsequent section outlines the methodology employed in conducting the research. Following this, the fourth section presents the results and analysis of all the proposed HES. Finally, the last component of the paper offers a comprehensive discussion of the study's conclusions along with some recommendations.

## 2 Proposed system description and modeling

**Fig. 1:**
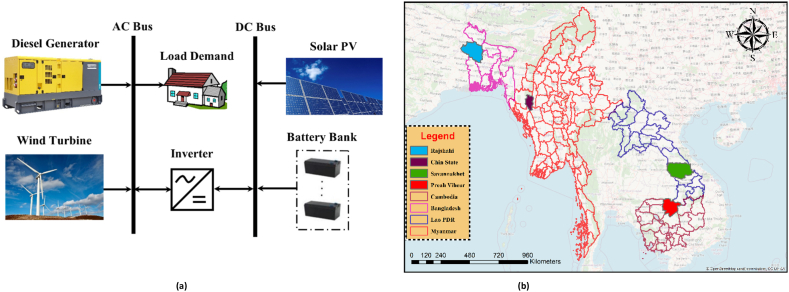
(a). Schematic of proposed HES for implementation in four distinct locations, (b). Study area in four different locations.

The arrangement of the HES system is illustrated in Fig. 1a. The majority of sources of clean energy consist of solar photovoltaic and wind power, supplemented by a backup system comprising a diesel engine and battery bank to maintain an uninterrupted supply of energy. The HES is responsible for the generation, conversion, distribution, and storage of electricity. The bidirectional converter is interconnected with both the direct and alternative current buses in this operation, while battery storage is utilized to store surplus power generated by renewable energy sources. The utilization of diesel generators serves the purpose of enhancing the dependability of the electrical grid during periods of high demand as well as in situations where battery storage and renewable energy source capacity systems fall short of meeting the required power supply.

### 2.1 Input data

#### 2.1.1 Study area, load demand, and renewable energy resource data

As illustrated in Fig. 1b, the study region encompasses Cambodia, Laos, Myanmar, and Bangladesh, which are recognized as the four least developed states in Asia. The geographical coordinates of Antil village in Choam Ksant District, Preah Vihear Province, Cambodia, are situated at 14°3.8′N and 104°35.4′E. The community consists of 160 dwellings and a total population of 720 individuals. Ban Thongpang village is located at coordinates 16°9.9′N and 105°41.6′E within the Thapangthong District of Savannakhet Province, situated in Laos. The population of this village comprises 196 families and a total of 1216 residents. Muitui village is situated inside the administrative boundaries of Mindat Township, located in Chin State, Myanmar, precisely at coordinates 21°25.8′N and 93°55.1′E. Within the confines of this particular hamlet, there exists a total of one hundred individual dwellings, which collectively accommodate a population of four hundred and ninety individuals.

**Fig. 2:**
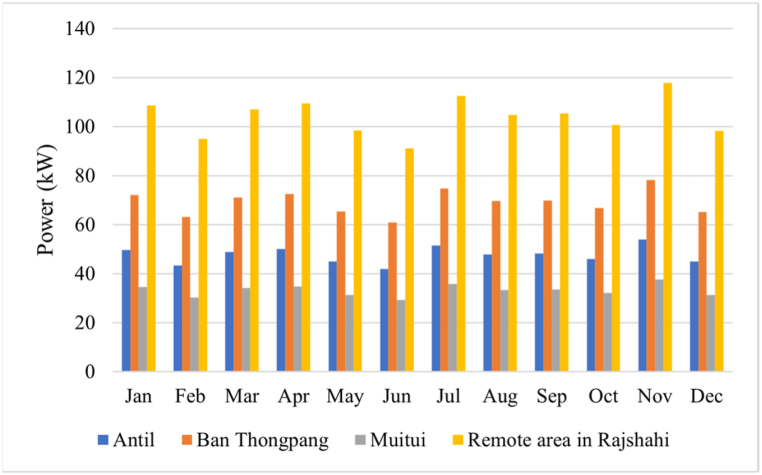
Monthly load consumption in four different locations.

The assessment of the electrical demand from end-users and the selection of the most authentic model for electrical consumption are key focal points in this research. The framework has been specifically built to effectively handle a higher peak power demand, hence ensuring compliance with the electrical load criteria. The design of load characteristics is contingent upon the income level and socioeconomic position of individual countries, with slight variations in load needs. This report presents a categorization of four distinct geographical regions that encompass the least developed nations in Asia. The villages of Antil, Ban Thongpang, Muitui, and a remote area in Rajshahi collectively consumed a daily average of 363.26 kWh, 521.59 kWh, 204.49 kWh, and 627.68 kWh, respectively. The peak power demand in these areas was recorded at 53.85 kW, 78.23 kW, 37.54 kW, and 117.82 kW, respectively. The monthly energy use is illustrated in Fig. 2. To enhance the authenticity of load demand data, the daily electrical load patterns are first described and subsequently randomized. For the purpose of this study, random rate variations of 10% and 20% will be employed on a daily and time-step basis.

**Fig. 3:**
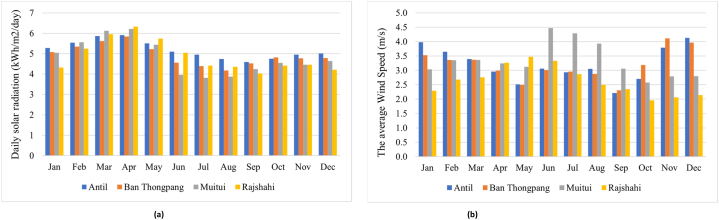
(a). Monthly solar irradiation for distinct four locations, (b). Monthly wind speed for distinct four locations.

Solar energy consumption is most favorable in four specific geographical places due to their ability to consistently collect solar radiation throughout the entire year. The data shown in Table 1 pertains to the geographical characteristics, meteorological conditions, and energy consumption patterns of the regions under investigation. Antil village has the highest average solar irradiations, measuring at 5.18 kWh/m^2^/day, when compared to Ban Thongpang village (4.93 kWh/m^2^/day), a distant region in Rajshahi (4.88 kWh/m^2^/day), and Muitui village (4.83 kWh/m^2^/day). On the other hand, it is noteworthy that Rajshahi, a remote area, exhibits the greatest clarity score of 0.55, with Antil village (0.54), Muitui village (0.53), and Ban Thongpang (0.52) following suit. Fig. 3 illustrates the mean monthly solar irradiance and wind velocity for the four separate locations. In 2023, meteorological data for multiple study areas was made by NASA through the use of the HOMER. This data included information on solar irradiation, specifically the average monthly values spanning a period of 22 years from 1983 to 2005. Data on wind speed was provided, with average monthly values derived from a 10-years period between 1983 and 1993.

**Table 1:** Geography, meteorology, and energy demand for study locations.

Study location	Antil area, Cambodia	Ban Thongpang region, Laos	Muitui region, Myanmar	Remote area in Rajshahi, Bangladesh
Geographical coordination	14°3.8′N 104°35.4′E	16°9.9′N 105°41.6′E	21°25.8′N93°55.1′E	24°22.5′N 88°29.8′E
Average solar irradiation (kWh/m^2^/day)	5.18	4.93	4.83	4.88
Average clearness index	0.54	0.52	0.53	0.55
Average wind speed (m/s)	3.20	3.18	3.34	2.64
Energy demand (kW)	363.26	521.59	204.49	627.68
Peak power (kW)	53.85	78.23	37.54	117.82

April has the maximum degree of solar irradiation absorption, while September experiences the lowest level, as depicted in Fig. 3a. From November to April, the rural regions of Rajshahi had the highest levels of solar irradiation, measuring at 6.33 kWh/m^2^/day. This value surpasses the solar irradiance seen in Antil village (5.91 kWh/m^2^/day), Ban Thongpang village (5.84 kWh/m^2^/day), and Muitui village (6.21 kWh/m^2^/day). Muitui experiences the lowest solar irradiance, measuring at 3.82 kWh/m^2^/day, between the rainy season, which covers from May to September. This level of solar irradiance is similar to that observed in Antil, Ban Thongpang, and the remote Rajshahi regions. Antil experiences a higher level of solar irradiation during the rainy season in comparison to other places. Furthermore, it is worth noting that the solar irradiation in Antil village exhibits minimal variation between the dry and rainy seasons, thereby creating a favorable environment for academic pursuits. The average wind speeds in the several study regions are illustrated in Fig. 3b. The Muitui region exhibits comparatively elevated wind velocities of 3.34 m/s in comparison to the remaining regions. On the other hand, it has been noted that the wind speed in remote communities in Rajshahi is comparatively lower, measuring at 2.64 m/s, in comparison to other places.

#### 2.1.2 Economic and technical inputs

The project's timeframe was established as a period of twenty-five years. The economic analysis [8,35,36] used nominal discount rates of 7.5%, 8.3%, 5.9%, and 10% for Cambodia, Laos, Myanmar, and Bangladesh, respectively. Additionally, inflation rates of 2.3%, 0.1%, 4%, and 2% were taken into account for the aforementioned countries. Table 2 presents an overview of the technical specifications and corresponding financial expenses associated with the primary components of the HES. It is worth noting that the capitals, replacements, and operations and maintenances expenditure for both Cambodia and Laos are computed in an identical manner. Furthermore, Table 2 presents a breakdown of expenses related to capital expenditures, replacements, and operations and maintenance. These costs are categorized into three unique values, each representing the component costs specific to Cambodia-Laos, Myanmar, and Bangladesh. Furthermore, the currency used for the purposes of this study is the United States Dollar (USD), which is represented by the dollar symbol ($).

**Table 2:** Technical specifications and financial cost of the major components of HES.

Component and sources	Characteristics	Values
PV module [8,[35], [36], [37], [38], [39], [40]]	Nominal power	327 W
	Panel efficiency	20.4 %
	Rated voltage	54.7 V
	Rated current	5.98 A
	Derating factor	88 %
	Lifetime	25 Years
	Capital cost	1000; 1560; and 1300 $/kW
	O&M cost	14, 10, and 13 $/kW/y
Wind turbine	Rated capacity	5.1 kW
[8,[35], [36], [37], [38], [39], [40]]	Cut-in velocity	2.7 m/s
	Rated wind velocity	11 m/s
	Cut-out velocity	55 m/s
	Hub height	15 m
	Rotor diameter	5.24 m
	Lifetime	20 Years
	Capital cost	1700; 1700; and 2300 $/kW
	O&M cost	35, 39, and 69 $/kW/y
Battery bank	Nominal capacity	6.91 kWh
[8,16,37,39,40]	Nominal voltage	6 V
	Roundtrip efficiency	80 %
	Lifetime	10 Years
	Capital cost	1100; 800; and 1100 $/kWh
	Replacement cost	1100; 800; and 1100 $/kWh
	O&M cost	10, 10, and 10 $/y
Diesel generator	Nominal capacity	25, 50, and 100 kW
[8,37,39,40]	Lifetime	15,000 h
	Capital cost	500, 400, and 370 $/kW
	Replacement cost	500, 400, and 370 $/kW
	O&M cost	0.03, 0.05, and 0.05 $/h/kW
Inverter	Rated power	1 kW
[8,37,40,41]	Conversion efficiency	95 %
	Capital cost	750, 500, and 750 $/kW
	Replacement cost	750, 500, and 750 $/kW
	O&M cost	15, 15, and 15 $/kW/y

### 2.2 The HES components modeling

The component characteristics inside a HES are crucial to ascertaining the optimal size for system configurations. The suggested HES comprises many components, including solar photovoltaic, wind energy as a clean energy source, a reserve diesel engine, a medium-storage battery bank, and a bidirectional converter.

#### 2.2.1 Photovoltaic modeling

For this study, the researchers selected a monocrystalline PV module with an efficiency of 20.4% and a power output of 327 W. The photovoltaic (PV) modules possess 54.7 V of a rating voltage, 5.98 A of a rating current, a de-rating factor of 88%, and a temperature coefficient for power of 0.35% per degree Celsius. In light of the relatively limited longevity of solar photovoltaic (PV) modules, a project duration of 25 years has been selected. The PV modules' power output may be calculated using Eq. (1), as described in Refs. [32,40].

(1) $$P_{PV}\left(t\right)=R_{PV}D_{PV}\left(\frac{G_{PV}\left(t\right)}{G_{ref}}\right)\left[1+\alpha_{p}\left(T_{PV}\left(t\right)-T_{ref}\right)\right]$$

Where R_PV_ refers to PV capacity rating (kW), D_PV_ is the de-rating factor (%), α_p_ is the powering temperature coefficient (%/°C), GPV represents the plane of array irradiance (kW/m^2^) on the module, G_ref_ indicates the plane of array irradiance under standard test conditions (STC), T_PV_ and T_ref_ refer to the module’s temperatures under operation and STC condition (°C), respectively. Derating factor is the decline in the PV module's power output caused by dust, wire losses, shade, and aging [42]. The PV module has a 25 years lifespan.

#### 2.2.2 Wind turbine modeling

Powered by wind turbine at any velocity V is denoted by Eq. (2) [40,43].

(2) $${\;P}_{WT}\left(t\right)\left\{ \begin{array}{c} 0,v\left(t\right)\leq v_{cut-in}\;or\;v\left(t\right)\geq v_{cut-out}\\ P_{r}\left(\frac{v^{3}\left(t\right)-v_{cut-in}^{3}}{v_{r}^{3}-v_{cut-in}^{3}}\right),v_{cut-in}<v\left(t\right)<v_{r}\\ P_{r},v_{r}\leq v\left(t\right)<v_{cut-out} \end{array}\right.$$

Where P_r_ represents the power rating of the turbine, V_cut-out,_ V_cut-in_, and V_r_ refer to the cut-out velocity, cut-in velocity, and rating velocity, respectively. The wind turbines have a lifespan of twenty years.

The hub height of the wind turbine directly affects both the wind velocity and energy output. Furthermore, it should be noted that the measured height of the hubs exhibits variation due to factors such as the specific wind turbine model and the prevailing wind speed at different elevations. The calculation of H is determined by utilizing the powering formula, as depicted in Eq. (3) [44]. In this equation, V_ref_ denotes the measurement of the reference wind speed obtained at the reference hub level, H_ref_. Additionally, δ represents the powering exponents formula, or Hellmann exponents, which have a range of values from 0.10 to 0.25 [45].

(3) $$v\left(t\right)=v_{ref}\left(t\right)\times {\left(\frac{H}{H_{ref}}\right)}^{\delta }$$

#### 2.2.3 Diesel generator modeling

For this study, diesel generators with power outputs of 25 kW, 50 kW, 75 kW, 100 kW, and 125 kW have been used. The HOMER software assumes a linear fuel utilization trend for diesel generators. The operational lifespan of the diesel generator has been predetermined to be 15,000 h. The projected fuel prices in various villages are as follows: 1.15 dollars per liter in Antil village, 1.15 dollars per liter in Ban-Thongpang village, 1 dollar per liter in Muitui village, and 0.80 dollars per liter in the remote Rajshahi village. The fuel utilization of the diesel engine is calculated by Eq. (4), as stated in Refs. [8,46].

(4) $$F_{DG}\left(t\right)=aR_{DG}+bP_{DG}\left(t\right)$$

Where a is the coefficient due to fuel curve intercepting, b is the fuel curve coefficient's slope as a function of diesel generator capacity, R_DG_ is the diesel engine's capacity rating, and P_DG_ is the powering production for any given time period.

#### 2.2.4 Battery storage modeling

This study utilizes lead-acid batteries because of their ability to absorb excess energy during the charging process and then supply energy when there is an inadequate supply from renewable energy sources to meet demand requirements. The upper limit of electrical power that a battery system may intake, as established by Eq. (5) [47]. The determination of the maximum discharging capacity of a battery can be achieved by employing Eq. (6), as referenced in the source [47].

(5) $$P_{bc}\left(t\right)=\frac{kQ_{s}\left(t\right)e^{-k}+Q\left(t\right)kc\left(1-e^{-k\Delta t}\right)}{1-e^{-k\Delta t}+c\left(k\Delta t-1+e^{-k\Delta t}\right)}$$

(6) $$P_{bd}\left(t\right)=\frac{-k{Q{s^{-k\Delta t}}^{-k\Delta t}}_{\max}}{1-e^{-k\Delta t}+c\left(k\Delta t-1+e^{-k\Delta t}\right)}$$

Where Q_max_ denotes the storage's entire capacity, Q_s_(t) represents the accessible power at the beginning of the time interval that is greater than the minimum states of charges level, Q(t) represents the overall electricity power at the beginning of the duration period interval, c represents the storages capacities utilization, k represents a fixed value for the storage capacity, and Δt represents the duration of time to the time t. To optimize the longevity of the battery, it was determined that the minimum amount of battery charge should be set at 20% [3,8]. The longevity of a battery is quantified in terms of either years or cycles, which are determined through the process of charging and discharging. The current study examines the duration of time that has elapsed since the publication of the literary source [48]. The battery exhibits a longevity of 10 years and a total energy production of 6,879,60 kW-hours.

#### 2.2.5 Bidirectional converter modeling

The bidirectional converter facilitates the transformation between direct currents (DC) and alternating currents (AC). DC power is comprised of solar and battery electricity that has been transformed into AC power in order to fulfil electrical consumption needs. Conversely, AC power generated from wind sources is converted into DC power for the purpose of charging the battery. In HOMER, the converter's capacity is automatically defined by taking into account the bus system's maximum power transfer. The inverter's power outputs are determined by Eq. (7). The estimated operational duration of the bidirectional converter is 15 years.

(7) $$P_{out}=P_{in}\times \eta_{inv}$$

Where P_in_ is the inverter’s power input, and η_inv_ is the inverter’s efficiency.

## 3 Methodology

### 3.1 HOMER simulation criteria

The economic analysis of potential system designs is a crucial component in this particular research, as it aims to achieve cost-effectiveness while ensuring the reliability of the HES system. To ascertain the financial advantages, it is imperative to appraise not solely the operational expenditures but also the initial outlay and subsequent replacement charges associated with the system. The net present cost (NPC) encompasses all expenses accrued during the entirety of a system's lifespan [49,50]. The HOMER framework employed the NPC and COE as optimization criteria to categorize various configurations under different circumstances and determine the most cost-effective configuration. The non-player character NPC can be determined by utilizing Eq. (8) as referenced in the sources [8,51].

(8) $$C_{NPC}=\frac{C_{TAC}}{f}$$

Where C_TAC_ represents the overall annualizes costs, f is the capital recoveries factors calculated by Eq. (9) [46,52] and The COE is calculated utilizing Eq. (10) [51].

(9) $$f=\frac{i{\left(1+i\right)}^{n}}{{\left(1+i\right)}^{n}-1}$$

Where n refers to the total number of years, i refers to the annualizes realistic interest rate (Discount rate), and E_served_ is the total electrical load served.

(10) $$COE=\frac{C_{TAC}}{E_{served}}$$

### 3.2 HOMER optimization

This study utilizes the HOMER software tool to ascertain the most favorable metric for each component, taking into consideration the least NPC and COE. The process operates in three distinct steps. During the preliminary stage, it is imperative to carefully choose the specific areas of study, ascertain the electricity consumption requirements, and gather meteorological data pertaining to sun irradiance, wind speed, and temperatures within the designated study regions. In order to utilize HOMER effectively, it is necessary to input the technological characteristics and financial data pertaining to the key component of the HES, as outlined in Table 2. During the second stage, HOMER undertakes optimization and analysis of the given data, afterwards producing a compilation of feasible system combinations based on a decreased net present cost (NPC). In this scenario, every system configuration is designed with the objective of fulfilling a certain electrical demand requirement while also taking into account additional technical factors such as surplus energies, the fraction of renewable energy (RF), and capacity deficits. Finally, the aforementioned results will be evaluated and contrasted using technical, economic, and performance metrics, including excess energy, renewable fraction, and capacity shortage. HOMER utilizes a range of operating processes to enhance system configurations for optimal performance.

### 3.3 Simulation setting

In HOMER, load following (LF) and cycle charge (CC) are dispatching procedures that play a significant role in the management of generator and battery storage operations in situations where there is insufficient power to satisfy the load consumption. By employing the load-following method, the generator operates at a capacity that precisely matches the prevailing power requirements, hence abstaining from charging the batteries. By employing the cycle charge method, a generator can function at or near its maximum capacity, thereby utilizing any surplus energy to charge the battery. This study examined the load following approach. This approach involves the comparison of power generated by solar and wind sources with the capacity requirement. In the event that there is surplus energy beyond the requirements of the electrical power demand, it is allocated towards the charging of batteries until they attain their maximum capacity, subsequently leading to their discharge. In the event of an electrical shortage, the battery could be discharged to satisfy the electrical load usage if there is enough battery charge available. Alternatively, if the battery charge is insufficient, the diesel engine will be used in order to satisfy the electrical consumptions.

### 3.4 The multi-criteria decision

**Fig. 4:**
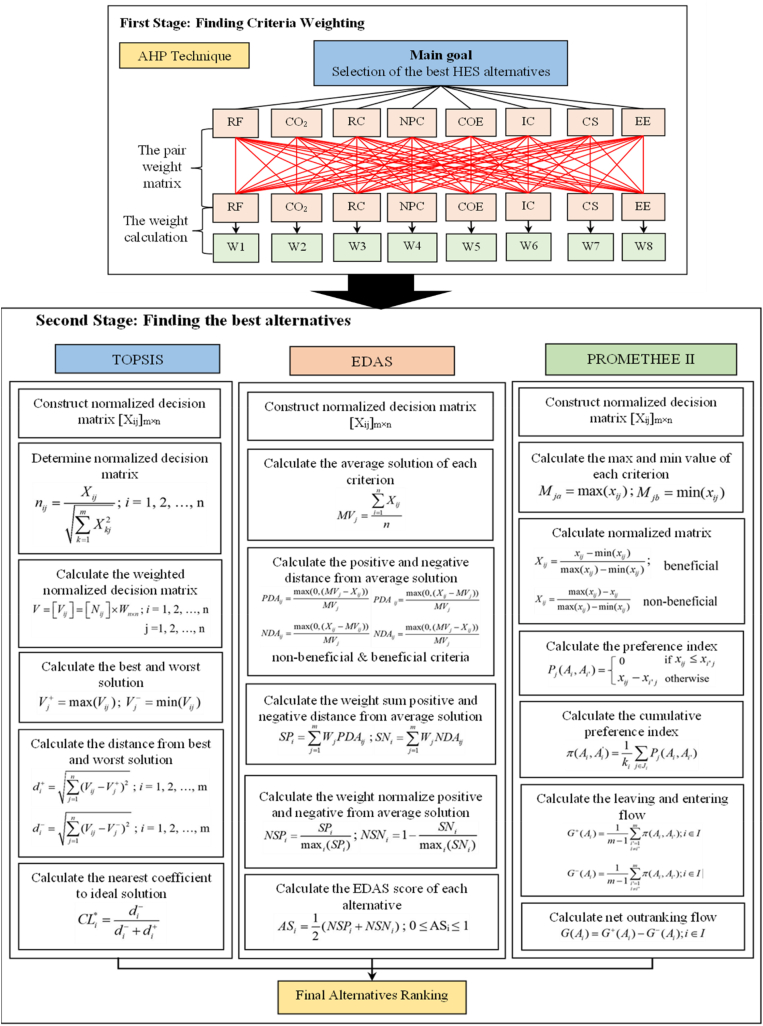
The structure of the designed hybrid MCDM method.

Assessing the optimal suitable HES designs from a wide range of possibilities presents a complicated decision-making endeavor that encompasses several sustainability factors and competing criteria. Defining a global optimum that effectively optimizes all criteria in a simultaneous manner is a highly challenging task [53]. The MCDM method is proposed as a feasible approach for addressing this problem and determining the most optimal solutions from the optimization outcomes derived from HOMER. This is attributed to its intrinsic ability to assess several alternatives by considering the relative importance of objectives [54]. The present study introduces a two-stage Multiple Criteria Decision Making (MCDM) framework, as illustrated in Fig. 4. During the initial phase, the AHP is employed to ascertain the corresponding weight of various performance criteria. During the second phase, the ideal compromise configuration of a hybrid energy system (HES) among many feasible alternatives is determined using three ranking MCDM methods, which are TOPSIS, EDAS, and PROMETHEE II.

#### 3.4.1 The criteria of MCDM method

Prior to determining the most suitable arrangement for meeting the electricity requirements in Asia's least developed countries (LDCs), it is imperative to assess the factors that impact the decision-making process. Various elements, including environmental, economic, and technological considerations, along with the distinctive qualities, incentives, and regulatory measures pertaining to the case study's development of renewable energy utilization, can potentially impact these criteria. The investigation's findings indicate that a total of eight criteria were selected. The following terms will be used in this academic discussion: (1) renewable fraction (RF), (2) CO2 emission (CO2), (3) renewable capacity (RC), (4) net present costs (NPC), (5) energy costs (COE), (6) initial capital costs (IC), (7) capacity shortage (CS), and (8) excess electricity (EE).

The primary objectives of the MCDM model encompass reducing the net present cost (NPC) throughout the project's duration, minimizing COE required to fulfill the demand consumption, decreasing the IC, mitigating carbon dioxide (CO_2_) emissions into the atmosphere, minimizing the IC, achieving EE after meeting the load requirements, and maximizing the environmental friendliness of electricity generation through sustainable renewable energy sources, as indicated by higher values of RF and RC. Furthermore, the aforementioned characteristics were assessed via the Analytic Hierarchy Process (AHP) methodology.

#### 3.4.2 The AHP approach

The AHP is a widely recognized the MCDM process that was originally proposed by Thomas L. Saatty. The AHP is a MCDM technique that involves making pairwise comparisons. It has been extensively employed by researchers in several domains [34,55]. The modeling technique encompasses the procedures illustrated in Fig. 4. To begin, it must be to develop hierarchy structure for the given matter. In order to develop a hierarchy structures, it is necessary to carefully examine and interrelate the many aspects within each level, taking into account both the higher and lower levels. Broadly speaking, the primary level pertains to the purpose, the secondary level pertains to the criteria for periodization of the alternatives, and the tertiary level pertains to the verification option. The task at hand involves the computation of paired matrices and the determination of the corresponding weights for each condition. In accordance with the prescribed hierarchical framework, the computation of the pairwise matrices necessitate the exercise of judgement by the decision-makers. The final phase involves the determination and validation of the consistency ratio (CR). The evaluation of judgements is facilitated by verifying consistency through the comparison of pairwise decisions. In this particular scenario, it is advisable to conduct a repeated pairwise comparison. It is advisable to maintain a CR (confidence level) below 10% in order to ensure that significant findings are obtained. Values beyond 10% are deemed unsuitable for acquiring meaningful outcomes. The assessment of the consistency index (CI) involves determining the reciprocal matrix A that possesses the highest Eigenvalue, with n being the size of the matrix. Furthermore, the consistency ratio (CR) serves as an approximation of the matrix's consistency index (RI), which is subject to variation based on the matrix's size.

#### 3.4.3 The TOPSIS approach

TOPSIS is well recognized as a highly utilized, efficient, and well-regarded MCDM approach. This approach to discontinuous multiple-criteria evaluation involves the assessment of m potential alternatives in relation to n criteria, resulting in the ranking of these configurations based on their alignment with the desired option. The TOPSIS technique is defined as the assumption that the selected solution has the minimum geometrical distances to the positivity ideal solutions and the maximum geometrical distances to the negativity ideal solutions [34,56]. The modeling technique encompasses the procedures illustrated in Fig. 4. Construct a decision matrix denoted as [X_ij_]_m×n_, where m represents the number of alternatives and n represents the number of criteria. Next, employ the normalization approach to compute the normalized decision matrix. Determine the normalized weighted decision matrix.

Next, it is necessary to select the most favorable and unfavorable alternatives. The ideal value for the positive criterion is determined by selecting the highest number assigned to the criteria in the weight normalization decisions matrix of separate possibilities. Conversely, the optimal value for the negative criterion is determined by selecting the lowest values. When determining the optimal negative solutions, this association exhibits an inverse relationship. Determine the magnitude of the disparity between the most favorable and least favorable outcomes. Determine the coefficient (CL*) of the nearest selection to the optimal solutions. Ultimately, the alternatives are organized based on their CL* values, whereby any option possessing a higher CL* is selected.

#### 3.4.4 The EDAS approach

The EDAS technique is a promising approach that holds potential for application in the field of MCDM. The efficiency of EDAS has been proven to be comparable to that of TOPSIS. For instance, where decision-making is in contention. Both the EDAS and TOPSIS methods are capable of evaluating criteria by utilizing a decision matrix that consists of exact information. The approach subsequently assesses the nature of criteria that exert either a positive or negative influence on the decision-making process. Furthermore, it should be noted that both approaches necessitate the inclusion of weight as an input for the criterion. However, it is important to highlight that neither strategy has the capability to generate these weights autonomously. The determination of the optimal option in the EDAS framework involves the calculation of the range between the value of the typical option (MV) and the ideal solution, which is not explicitly computed. One of the main advantages of EDAS [34] is its tendency to yield a computing process that is simpler and more efficient compared to TOPSIS. The modeling technique encompasses the procedures illustrated in Fig. 4. Construct a decision matrix denoted as [X_ij_]_m×n_, where m refers to the number of alternatives and n rerefers to the number of criteria. Calculate the average solution for each criterion subsequently. Thirdly, it is necessary to ascertain the positive and negative attributes of the average solutions, denoted as PDA and NDA, respectively. Calculate the aggregate value obtained by multiplying the positive and negative distances from the mean solutions by their respective weights. Next, compute the weighted normalization of positivity and negativity based on the average solutions. The assessment score (AS) for each of the succeeding options will be determined, and then they will be sorted based on their AS. The option with the highest AS will be picked.

#### 3.4.5 The PROMETHEE II approach

PROMETHEE II is stated as follows [57,58] and shown in Fig. 4. First, create a decision matrix [X_ij_]_m×n_ with m alternatives and n criteria. Second, determine the minimum and maximum value of each criterion. Third, calculate the normalized matrices using linear normalization. Fourth, calculate the preference index, represented as P_j_(A_i_, A_i′_). Fifth, determine the cumulative preference index. Then, calculate the leaving and entering flow of each alternative. Finally, determine the net outranking flow (G(A_i_)) of each alternative, and then sort the options by G(A_i_); any alternative with a higher G(A_i_) is selected.

In conclusion, the final outcome reveals the relative ranking scores of each potential configuration of standalone hybrid energy systems (HES). After doing a comparative analysis of the three available raking processes, the optimal choice for supplying energy to Asia's LCS is determined.

## 4 Results and discussion

### 4.1 HOMER results

The HOMER software tool conducts simulations encompassing various configurations of wind energy, solar photovoltaic (PV) systems, diesel generators, and battery systems over four discrete geographical areas situated inside Asia's least developed countries. The evaluation of results includes the assessment of many factors, such as NPC, COE, RF, and EE, along with the determination of the most favorable component size. In this step, it should be noted that S1 denotes the configuration consisting of PV/DG/Bat, whilst S2, S3, S4, S5, S6, and S7 represent the configurations of PV/WT/DG/Bat, PV/Bat, WT/DG/Bat, Diesel, PV/WT/DG, and PV/WT/Bat, respectively.

#### 4.1.1 Case study 1, Antil village in Cambodia

Table 3 presents a comprehensive summary of the potential arrangements for the best outcomes inside the Cambodian community of Antil. This diesel-only system is the most expensive to generate electricity. The NPC and the initial capital cost are $1,132,988 and $25,000, respectively. The COE of this system is 0.611$/kWh, while the fuel consumption is 56,677 L. Consuming a massive amount of fuel results in the system emitting tons of carbon dioxide as well as other pollutant gases into the environment. On the other hand, PV/DG/Bat is the optimum scenario. The initial capital cost, the NPC, and the renewable fraction are $244,277, $476,216, and 89%, respectively. The COE is 0.257 $/kWh, while the fuel consumption is 5672 L. The amount of fuel consumed by PV/DG/Bat was lower compared to the diesel-only system, approximately; 10% of diesel fuel consumption as well as the carbon footprint can be deducted. The PV/GD/Bat configuration has a total of 94 kW of PV power, 50 kW of diesel generators, 608 kWh of reserve batteries, and 38 kW of bidirectional converters. The size of the bidirectional converter is dictated by the maximum power that is converted between the DC and AC buses in both directions.

**Table 3:** An overview of the optimization results for Antil village in Cambodia.

Configuration	PV (kW)	Wind (kW)	Diesel (kW)	Battery (kWh)	Converter(kW)	COE ($/kWh)	NPC ($)	IC ($)	CO2 (kg/y)	Diesel (L)	RF (%)	EE (kW/y)	CS (kW/y)
PV/DG/Bat	94	–	50	608	38	0.257	476,216	244,277	14,848	5672	89	13,446	5.42
PV/WT/DG/Bat	94	5.1	50	580	36	0.258	477,780	247,765	14,494	5537	89.3	15,573	5.15
PV/Bat	120	–	–	1796	47	0.412	763,249	441,295	–	–	100	37,707	127
PV/WT/DG	94	5.1	50	0	34	0.473	877,460	153,615	90,919	34,730	31.1	108,815	57.6
PV/WT/Bat	94	122.4	–	1465	52	0.479	886,754	574,419	–	–	100	53,963	132
WT/DG/Bat	–	91.8	50	138	11	0.518	959,272	211,384	94,839	36,228	21.7	3964	–
DG	–	–	50	–	0	0.611	1,132,988	25,000	148,373	56,677	–	22,074	59.4

**Fig. 5:**
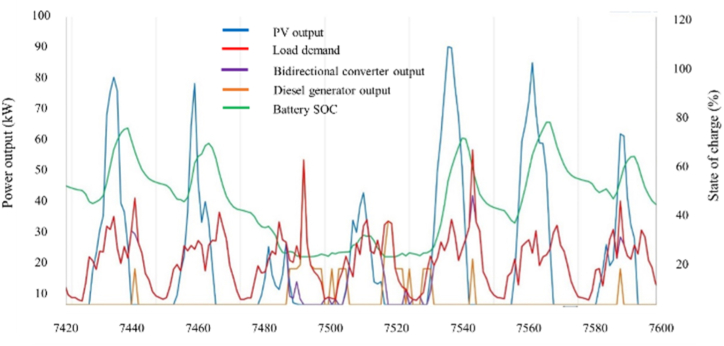
The statistic of a time series of energy mixing in dry season (November).

The energy composition data for each component is illustrated in Fig. 5. Peak demand often arises within the time frame of 6–8 p.m., a period characterized by the absence of solar energy. Furthermore, the demand capacity of the PV/DG/Bat arrangement is fulfilled by the batteries, facilitated by a bidirectional converter, and the diesel source, as illustrated in Fig. 5. Nevertheless, in instances where the amount of photovoltaic energy generated exceeds the energy consumption during daylight hours, the surplus energy is utilized for the purpose of recharging the batteries. Despite the fact that this particular scenario exhibits a greater photovoltaic capacity rating, bidirectional converters demonstrate a notable advantage in terms of system size, as they are considerably smaller. Furthermore, this perspective is substantiated by several studies [32,59,60].

By integrating a 5.1 kW wind turbine, the PV/DG/Bat configuration achieves a comparable scale to the PV/WT/DG/Bat alternative. However, the latter option has a notably higher NPC of $477,780 and a COE of $0.258 per kilowatt-hour. The insufficiency of wind turbines in contributing to energy generation and the subsequent increase in prices can be attributed to the reduced wind speeds observed in Antil village. Both systems exhibit comparable levels of radio frequency energy generation, ranging from 89% to 89.3%. Additionally, they produce a surplus of power, with an annual range of 13,446 kWh to 15,573 kWh. Based on reference [61], it was revealed that the examination of the PV/DG/Bat configuration resulted in 0.302 $/kWh of COE, demonstrating similarity to the results of other contemporary research. Depending on Hossain et al. [62], the PV/Diesel/Batteries system studied in Malaysian case studies demonstrates a COE of 0.28 USD per kilowatt-hour. As a result, it could be demonstrated that the PV/DG/Bat combination exhibits a lower COE of 0.257 $/kWh compared to the PV/Bat design, which has a 0.412 $/kWh COE, as well as other configurations.

The results show that PV/DG/Bat is the optimal configuration to reduce the impact of global warming and greenhouse gas emissions by approximately 10% that occur from electricity production compared to a diesel-only system, while the cost of electricity is reduced.

**Fig. 6:**
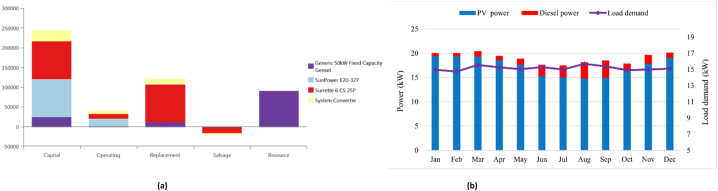
(a). An overview costs of the PV/Diesel/Battery option, (b). Monthly average energy sharing for meeting load demands of PV/Diesel/Battery configuration for Antil village in Cambodia.

Fig. 6a illustrates a comprehensive overview of the components associated with the PV/DG/Bat option's cost. The battery necessitates the highest level of capital investment, with the PV modules ranking second in terms of financial requirements. While the expenses associated with replacing backup battery systems are higher during the duration of the project, it is crucial to notice that the cost of resources, particularly gasoline prices, far exceeds the initial investment. As illustrated in Fig. 6b–a diesel generator exhibits a higher energy output in the presence of substantial electrical demand. The average monthly energy production from photovoltaic (PV) panels experiences a decrease during the rainy season in comparison to the dry season, primarily due to continuous rainfall and cloud convergence.

#### 4.1.2 Case study 3, Ban Thongpang village in Laos

**Fig. 7:**
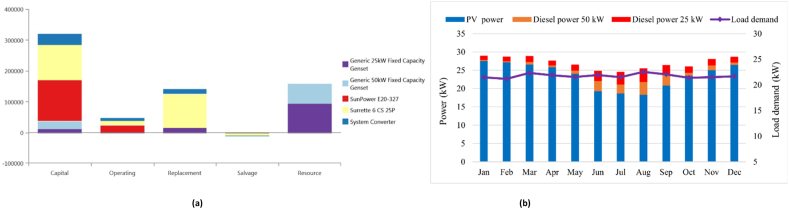
(a). An overview costs of the PV/Diesel/Battery option, (b). Monthly average energy sharing for meeting load demands of PV/Diesel/Battery configuration for Ban Thongpang village in Laos.

Table 4 presents a comprehensive summary of the potential arrangements for all the possible arrangements in the village of Ban Thongpang in Laos. The PV/WT/Bat configuration is the highest system to produce electricity. The initial capital cost is higher compared to other configurations due to the increased capacity of the battery backup system, but this system is environmentally friendly and doesn’t emit carbon dioxide into the atmosphere. The COE, the NPC, and the renewable fraction of this system are 0.553 $/kWh, $1,359,568, and 100%, respectively. After that, the diesel-only system is the scenario that emits the most carbon dioxide emissions into the atmosphere. The diesel fuel consumption of this system is 63,983 L, while the COE is 0.454 $/kWh. In contrast, the PV/DG/Bat system is the best configuration compared to other scenarios. The COE, the NPC, and the renewable fraction of this system are 0.265 $/kWh, $653,235, and 84.1%, respectively. The initial capital cost is $318,931, while the diesel fuel consumption is 10,690 L. Compared to the diesel-only system, the carbon dioxide emissions are reduced by almost 16.70%. The NPC and COE for photovoltaic (PV) systems combined with diesel generators and batteries (PV/DG/Bat) are similar to those of PV systems combined with wind turbines, diesel generators, and batteries (PV/WT/DG/Bat). The systems comprising PV/DG/Bat, PV/WT/DG/Bat, and PV/WT/Bat exhibit renewable fractions of 84.1%, 84.3%, and 100%, respectively. The initial capital cost and the NPC of the PV/Bat system are higher, $718,526, and $1,201,356, respectively, due to the need for more capacity in the battery backup system. Additionally, the overview cost of PV/DG/Bat configuration is depicted in Fig. 7a. Fig. 7b illustrates the mean monthly allocation of energy from different sources in order to meet the requirements of electrical consumption.

**Table 4:** An overview of the optimization results for Ban Thongpang village in Laos.

Configuration	PV (kW)	Wind (kW)	Diesel (kW)	Battery (kWh)	Converter(kW)	COE ($/kWh)	NPC ($)	IC ($)	CO2 (kg/y)	Diesel (L)	RF (%)	EE (kW/y)	CS (kW/y)
PV/DG/Bat	132	–	75	719	46	0.265	653,235	318,931	27,985	10,690	84.1	20,094	5.51
PV/WT/DG/Bat	132	5.1	75	691	48	0.267	656,689	322,888	27,717	10,588	84.3	22,197	5.22
PV/WT/DG	132	5.1	75	–	28	0.395	972,240	198,912	110,579	42,240	36.8	134,146	54.40
WT/DG/Bat	–	40.8	75	55	9	0.432	1,064,267	122,300	145,934	55,745	7.26	280	–
DG	–	–	75	–	–	0.454	1,116,935	37,500	167,498	63,983	–	3804	56.40
PV/Bat	190	–	–	2930	83	0.488	1,201,356	718,526	–	–	100	73,367	183
PV/WT/Bat	132	234.6	–	2073	90	0.553	1,359,568	928,047	–	–	100	92,189	181

The finding indicated that PV/DG/Bat is the suitable solution for lowering the greenhouse gas emissions by approximately 16.7% that occur from electricity production compared to a diesel-only system, while the cost of electricity is reduced.

#### 4.1.3 Case study 3, Muitui village in Myanmar

**Fig. 8:**
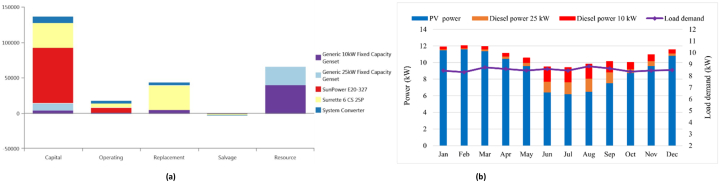
(a). An overview costs of the PV/Diesel/Battery option, (b). Monthly average energy sharing for meeting load demands of PV/Diesel/Battery for Muitui village in Myanmar.

Table 5 presents the potential arrangement of possible arrangements in Muitui village, located in Myanmar. The PV/Bat system is the most expensive system to generate electricity. The initial capital cost and NPC is higher compared to other configurations due to the increased capacity of the battery backup system, however, this system doesn't release carbon dioxide into the atmosphere, which makes it environmentally friendly. The COE, the NPC, and the initial capital cost of this system are 0.490 $/kWh, $472,742, and $305,502, respectively. Then, the diesel-only is the system that release the most carbon dioxide emissions into the atmosphere. The diesel fuel consumption of this system is 69,490 L, while the COE is 0.420 $/kWh. However, the hybrid PV/DG/Bat system is the optimum scenario compared to other scenarios. The COE, the NPC, the renewable fraction of this system are 0.269 $/kWh, $259,084, and 81.6%, respectively. The initial capital cost is $136,235, while the diesel fuel consumption is 5136 L. Comparing to the diesel-only system, the carbon dioxide emission is reduced almost 19.26%. The PV/GD/Bat scenario has a total of 50 kW of PV power, 35 kW of diesel generators, 304 kWh of reserve batteries, and 18 kW of bidirectional converters. Otherwise, hybrid PV/WT/DG/Bat, PV/WT/DG, and WT/DG/Bat systems ranks as second, third, and fourth, respectively. The COE of hybrid PV/WT/DG/Bat, PV/WT/DG, and WT/DG/Bat systems are $0.271, $0.361, and $0.399 as well. Fig. 8a illustrates the financial overview of the PV/DG/Bat option for Muitui village. Batteries have comparatively elevated expenses for replacement in relation to other types of equipment. As illustrated in Fig. 8b, an increased power consumption requires an augmented reliance on the diesel generator.

**Table 5:** An overview of the optimization results for Muitui village in Myanmar.

Configuration	PV (kW)	Wind (kW)	Diesel (kW)	Battery (kWh)	Converter(kW)	COE ($/kWh)	NPC ($)	IC ($)	CO2 (kg/y)	Diesel (L)	RF (%)	EE (kW/y)	CS (kW/y)
PV/DG/Bat	50	–	35	304	18	0.269	259,084	136,235	13,427	5136	81.6	8366	3.64
PV/WT/DG/Bat	50	5.1	35	276	17	0.271	261,822	141,358	12,744	4874	82.6	10,326	3.44
PV/WT/DG	50	5.1	35	–	15	0.361	348,241	108,389	40,441	15,464	42.5	49,099	35.20
WT/DG/Bat	–	20.4	35	28	3	0.399	385,218	53,379	58,847	22,510	11.2	267	4.83
PV/WT/Bat	50	56.1	–	774	36	0.418	402,570	281,107	–	–	100	21,534	74.10
DG	–	–	35	–	–	0.420	405,222	14,000	69,490	26,657	–	1084	39.50
PV/Bat	85	–	–	1327	38	0.490	472,742	305,502	–	–	100	47,173	72.8

The outcome show that PV/DG/Bat is the optimal solution for reducing the impact of global warming and greenhouse gas emissions by approximately 19.26% that occur from electricity production compared to a diesel-only system, while the cost of electricity is reduced.

#### 4.1.4 Case study 4, Rajshahi in Bangladesh

**Fig. 9:**
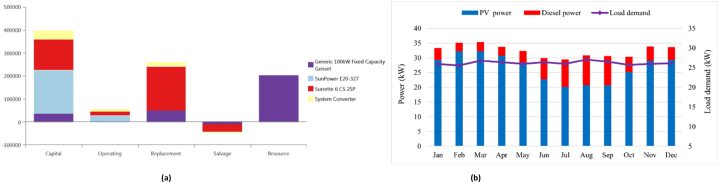
(a). An overview costs of the PV/Diesel/Battery option, (b). Monthly average energy sharing for meeting load demands of PV/Diesel/Battery for remote area in Rajshahi in Bangladesh.

Table 6 illustrates the potential arrangement of best outcomes in the rural community in Rajshahi in Bangladesh. The PV/WT/Bat configuration is the highest system to produce electricity. In this case is similar to case study 2 in Ban Thongpang inLaos. The COE, the NPC, and renewable fraction of this system are 1.010 $/kWh, $3,244,959, and 100%, respectively. After that, the diesel-only system ranks as third based on the COE and the NPC. The diesel fuel consumption of this system is 76,612 L, while the COE is 0.340 $/kWh. In contrast, the PV/DG/Bat system is the best configuration compared to other scenarios. The COE, the NPC, the renewable fraction of this system are 0.271 $/kWh, $869,020, and 77.3%, respectively. The initial capital cost is $396,506, while the diesel fuel consumption is 18,312 L. Comparing to the diesel-only system, the carbon dioxide emission is reduced almost 23.88%. The NPC and COE for photovoltaic (PV) systems combined with diesel generators and batteries (PV/DG/Bat) is similar to those of PV systems combined with wind turbines, diesel generators, and batteries (PV/WT/DG/Bat). The systems comprising PV/DG/Bat, PV/WT/DG/Bat, and PV/WT/Bat exhibit renewable fractions of 77.3%, 77.7%, and 100% respectively. The initial capital cost and the NPC of the PV/WT/Bat system are higher, $2,007,399, and $3,244,959, respectively, due to the need for more capacity in the battery backup system. Fig. 9a illustrates a comprehensive overview of the components associated with the PV/DG/Bat option's cost. This study needs to be observed that the proposed of the HES has a similar trend to Antil, Ban Thongpang, and Muitui in terms of requiring a greater capital cost for PV modules and resource cost. Fig. 9b illustrates the monthly power contributions from several sources in order to satisfy the electrical need.

**Table 6:** An overview of the optimization results for remote area in Rajshahi in Bangladesh.

Configuration	PV (kW)	Wind (kW)	Diesel (kW)	Battery (kWh)	Converter(kW)	COE ($/kWh)	NPC ($)	IC ($)	CO2 (kg/y)	Diesel (L)	RF (%)	EE (kW/y)	CS (kW/y)
PV/DG/Bat	145	–	100	829	51	0.271	869,020	396,506	47,879	18,312	77.3	23,509	12.8
PV/WT/DG/Bat	145	5.1	100	829	52	0.276	883,626	408,367	47,143	18,024	77.7	23,920	3.6
DG	–	–	125	–	–	0.340	1,089,927	46,250	200,422	76,612	–	2142	5.43
WT/DG/Bat	–	5.1	100	166	20	0.385	1,231,784	90,239	204,585	78,218	–	1815	20.4
PV/bat	230	–	–	3980	120	0.542	1,733,486	1,022,654	–	–	100	96,458	228
PV/WT/DG	145	295.8	100	–	48	0.674	2,157,120	941,793	143,163	54,735	30.7	230,652	229
PV/WT/Bat	145	571.2	–	2626	116	1.010	3,244,959	2,007,399	–	–	100	107,232	228

The finding indicated that PV/DG/Bat is the suitable solution for lowering the greenhouse gas emissions by approximately 23.88% that occur from electricity production compared to a diesel-only system, while the cost of electricity is reduced.

### 4.2 Selection of the best configuration for each study location using the MCDM

The utilization of MCDM methodologies can facilitate the identification and selection of the best viable configuration from the outcomes of the HOMER optimization process. The AHP, TOPSIS, EDAS, and PROMETHEE II methodologies were employed in this particular case to ascertain the most effective arrangement.

#### 4.2.1 The AHP technique for weighting criteria

The AHP is applied to determine the relative vital of all criteria by taking into account the judgements of decision-makers. Table 7 presents the pairwises comparison matrice of the AHP approach. Table 8 presents the relative significance of the weight criteria of the AHP technique as seen by decision-makers. The priorities of weight criteria for the Analytic Hierarchy Process (AHP) technique are presented in Table 8. The table reveals that the RF criterion holds the highest weight at 19.35%, followed by CO2 emission at 18.02%, NPC at 17.21%, CS at 14.74%, COE at 12.95%, RC at 9.67%, IC at 4.90%, and EE at 3.17%.

**Table 7:** The pairwise comparison matrix of the AHP technique.

Criteria	RF	CO_2_	RC	NPC	COE	IC	CS	EE
RF	1	1	2	1	2	5	1	7
CO_2_	1	1	2	1	2	3	1/2	9
RC	1/2	1/2	1	1/2	1/2	2	1	4
NPC	1	1	2	1	1	3	2	4
COE	1/2	1/2	2	1	1	3	1	3
IC	1/5	1/3	1/2	1/3	1/3	1	1/3	2
CS	1	2	1	1/2	1	3	1	3
EE	1/7	1/9	1/4	1/4	1/3	1/2	1/3	1

**Table 8:** Priority of weight criteria of AHP technique.

Criteria	RF	CO_2_	NPC	CS	COE	RC	IC	EE
%	19.35	18.02	17.21	14.74	12.95	9.67	4.9	3.17
Priority	1	2	3	4	5	6	7	8

#### 4.2.2 The TOPSIS, EDAS, and PROMETHEE II results

##### 4.2.2.1 Case study in Antil village in Cambodia

Within this particular portion, the use of three MCDM assessments, called TOPSIS, EDAS, and PROMETHEE II, is implemented to ascertain the most optimal compromise configuration for the Hybrid Energy System (HES) from a range of feasible possibilities. Table 9 displays the choices matrix of the assessment criteria versus the power configurations (alternatives) for the TOPSIS, EDAS, and PROMETHEE II methodologies. The calculation of several parameters in the TOPSIS method includes the normalized matrix, the weights normalized matrix, the better and least solution, the distance from the better and least solution, and the closest coefficient, as depicted in Fig. 4. The calculating process for the EDAS and PROMETHEE II methods is identical to that of the TOPSIS approach, as depicted in Fig. 4. Furthermore, Table 10 presents the measurements of the disparity between the optimal (di^+^) and suboptimal (di^−^) solutions, as well as the coefficient that closely approximates the ideal solution in the context of TOPSIS. Moreover, Table 10 presents the weights of normalized positivity (NSP_i_) and negativity (NSN_i_) derived from the average solutions, as well as the EDAS score (AS_i_). Finally, Table 10 presents a visual representation of the movement of individuals leaving (G^+^), arriving (G^−^), and outranking (G). It should be noted that S1 denotes the configuration consisting of PV/DG/Bat, whilst S2, S3, S4, S5, S6, and S7 represent the configurations of PV/WT/DG/Bat, PV/Bat, WT/DG/Bat, Diesel, PV/WT/DG, and PV/WT/Bat, respectively.

**Table 9:** Decision matrix of TOPSIS, EDAS, and PROMETHEE II including scenarios and criteria in Antil village in Cambodia.

Configuration	RF	CO_2_	RC	NPC	COE	IC	CS	EE
PV/DG/Bat (S1)	89	14,848	94	476,216	0.257	244,277	5.42	13,446
PV/WT/DG/Bat (S2)	89.3	14,494	99.1	477,780	0.258	247,765	5.15	15,573
PV/Bat (S3)	100	0	120	763,249	0.412	441,295	127	37,707
WT/DG/Bat (S4)	21.7	94,839	91.8	959,272	0.518	211,384	0	3964
DG (S5)	0	148,373	0	1,132,988	0.611	25,000	59.4	22,074
PV/WT/DG (S6)	31.1	90,919	99.1	877,460	0.473	153,615	57.6	108,815
PV/WT/Bat (S7)	100	0	226.4	886,754	0.479	574,419	132	53,963

**Table 10:** Overall evaluation of all configurations in Antil village using TOPSIS, EDAS, and PROMETHEE II in Antil village in Cambodia.

Method	TOPSIS				EDAS				PROMETHEE II			
Configuration	d_i_^+^	d_i_^-^	CL_i_*	Rank	NSP_i_	NSN_i_	AS_i_	Rank	G^+^	G^-^	G	Rank
PV/DG/Bat (S1)	0.044	0.192	0.8149	2	1	0.989	0.995	1	0.284	0.076	0.208	1
PV/WT/DG/Bat (S2)	0.044	0.193	0.8154	1	1	0.758	0.879	2	0.282	0.078	0.204	2
PV/Bat (S3)	0.182	0.176	0.4924	3	0.667	0.709	0.688	4	0.128	0.228	−0.099	6
WT/DG/Bat (S4)	0.232	0.120	0.3405	5	0.378	0.546	0.462	5	0.195	0.163	0.032	3
DG (S5)	0.296	0.065	0.1813	7	0.115	0	0.058	7	0.215	0.300	−0.085	5
PV/WT/DG (S6)	0.218	0.094	0.3012	6	0.043	0.644	0.344	6	0.148	0.186	−0.038	4
PV/WT/Bat (S7)	0.208	0.182	0.4668	4	0.825	0.615	0.720	3	0.086	0.308	−0.222	7

Based on the findings shown in Tables 10 and it can be observed that both EDAS and PROMETHEE II algorithms identified the PV/DG/Bat (S1) configuration as the most favorable choice. Conversely, the TOPSIS algorithm favored the PV/WT/DG (S2) configuration. The configuration consisting of PV/DG/Bat exhibits superior performance in terms of a better RF of 89%, fewer CO_2_ emissions of 14,848 kg per year, as well as lower values for the NPC and COE compared to alternative configurations.

##### 4.2.2.2 Case study in Ban Thongpang village (Laos), Muitui village (Myanmar), and remote area in Rajshahi (Bangladesh)

The evaluation criteria versus the alternative decisions matrix are presented in Table 11, incorporating the TOPSIS, EDAS, and PROMETHEE II methodologies. The procedure for determining the closest coefficient (TOPSIS), EDAS score (EDAS), and the outranking flow (PROMETHEE II) in Fig. 4 is identical to that of the initial case study conducted in Antil village. Based on the findings shown in Table 12, it can be observed that the PV/DG/Bat (S1) option was unanimously identified as the optimal configuration by all three approaches.

**Table 11:** Decision matrix of TOPSIS, EDAS, and PROMETHEE II including scenarios and criteria in Ban Thongpang village in Laos.

Configuration	RF	CO_2_	RC	NPC	COE	IC	CS	EE
PV/DG/Bat (S1)	84.1	27,985	132	653,235	0.265	318,931	5.51	20,094
PV/WT/DG/Bat (S2)	84.3	27,717	137.1	656,689	0.267	322,888	5.22	22,197
PV/Bat (S3)	100	0	190	1,201,356	0.488	718,526	183	73,367
WT/DG/Bat (S4)	7.26	145,934	40.8	1,064,267	0.432	122,300	0	280
DG (S5)	0	167,498	0	1,116,935	0.454	37,500	56.4	3804
PV/WT/DG (S6)	36.8	110,579	137.1	972,240	0.395	198,912	54.4	134,146
PV/WT/Bat (S7)	100	0	366.6	1,359,568	0.553	928,047	181	92,189

**Table 12:** Overall evaluation of all configurations in Ban Thonpang village in Laos using TOPSIS, EDAS, and PROMETHEE II.

Method	TOPSIS				EDAS				PROMETHEE II			
Configuration	d_i_^+^	d_i_^-^	CL_i_*	Rank	NSP_i_	NSN_i_	AS_i_	Rank	G^+^	G^-^	G	Rank
PV/DG/Bat (S1)	0.055	0.178	0.763	1	0.981	0.987	0.984	1	0.290	0.081	0.209	1
PV/WT/DG/Bat (S2)	0.056	0.178	0.760	2	0.979	0.637	0.808	2	0.288	0.082	0.205	2
PV/Bat (S3)	0.218	0.164	0.429	3	0.744	0.410	0.577	4	0.094	0.313	−0.218	6
WT/DG/Bat (S4)	0.225	0.112	0.332	6	0.456	0.212	0.334	6	0.246	0.146	0.100	3
DG (S5)	0.247	0.083	0.251	7	0.217	0	0.108	7	0.230	0.193	0.037	4
PV/WT/DG (S6)	0.186	0.103	0.356	5	0.138	0.679	0.409	5	0.179	0.165	0.014	5
PV/WT/Bat (S7)	0.238	0.175	0.423	4	1	0.289	0.644	3	0.075	0.422	−0.347	7

Additionally, the PV/WT/DG/Bat (S2) option emerged as the second-best alternative. The best configuration of Muitui and the isolated region of Rajshahi is equivalent to that of Ban Thongpang. The PV/DG/Bat (S1) option has been determined to be the most optimal choice across all three decision-making methods, namely TOPSIS, EDAS, and PROMETHEE II. The ideal configuration of PV/Diesel/Battery in Ban Thongpang, Muitui, and Rajshahi, respectively, exhibits greater reliability factor (RF) values of 84.1%, 81.6%, and 77.3%. Additionally, this configuration demonstrates reduced carbon dioxide (CO2) emissions of 27,985 kg/y, 13,427 kg/y, and 47,897 kg/y, respectively. Furthermore, it is associated with lower NPC and COE compared to alternative configurations.

#### 4.2.3 The environmental analysis of HES in different locations

The annual environmental emissions are caused by the amount of diesel generator fuel consumed annually. Table 3, Table 4, Table 5, and Table 6 demonstrated the annual CO_**2**_ emissions at various study sites in Asia's LDCs. In the absence of renewable energy and a reserve battery, diesel is utilized to fulfil load requirements. As a consequence, increased fuel use raises environmental degradation. As a result of the optimal configuration of each location, it determined that the PV/DG/Bat system in remote areas of Rajshahi consumes the most diesel fuel and has the lowest renewable fraction, generating 47,897 kg/y of CO_**2**_, followed by Ban Thongpang (27,985 kg/y), Antil (14,848 kg/y), and Muitui (13,427 kg/y). Moreover, compared to other system configurations, the diesel-only system in remote areas of Rajshahi requires the most diesel fuel and produces approximately 47,897 kg of CO_**2**_ emissions annually. In addition to CO_**2**_ emissions, UHC, CO, NO_**x**_, SO_**2**_ and PM emissions also affected the environment. Climate change and numerous health hazards are a result of the pollutants that have been produced. The environmental impact of the PV/WT/DG/Bat configuration at the research site is similar to that of the PV/DG/Bat configuration because of the reduced connection of wind power to the overall demand consumption. In addition, the PV/DG/Bat option produces substantially less emission than the other hybridization option. Clearly, the PV/DG/Bat scenario offers both economic and environmental benefits.

## 5 Conclusion

This study examined the viability of integrating solar PV, wind energy, and battery systems with an existing diesel generator using HOMER software in order to improve the current unstable electricity supply, reduce the effect of global warming caused by the production of electricity, and contribute to lowering costs in Cambodia, Laos, Myanmar, and Bangladesh. Furthermore, the present study proposed a novel approach that included the AHP, TOPSIS, EDAS, and PROMETHEE II methodologies used to verify all configurations obtained from HOMER’s results. Taking into account environmental, economic, and technical, the optimal configuration of HES was then determined using MCDM technique. The significant results of the research are shown as the following.

• The hybrid PV/DG/Bat configuration was the optimal solution for different locations such as Cambodia, Laos, Myanmar, and Bangladesh. The COE is 0.257 $/kWh, 0.265 $/kWh, 0.269 $/kWh, and 0.271 $/kWh, while the NPC is $476,216, $653,235, $259,084, and $869,020 for Cambodia, Laos, Myanmar, and Bangladesh, respectively.
• The MCDM results indicated that PV/DG/Bat provided an appropriate optimal scenario. This configuration was ranked first among the seven configurations in Cambodia, Laos, Myanmar, and Bangladesh, using TOPSIS, EDAS, and PROMETHEE II techniques. The renewable fraction is 89%, 84.1%, 81.6%, and 77.3% for Cambodia, Laos, Myanmar, and Bangladesh, respectively.
• The hybrid PV/DG/Bat system is the best configuration to reduce the impact of global warming and greenhouse gas emissions by approximately 10%, 16.7%, 19.26%, and 23.88% for Cambodia, Laos, Myanmar, and Bangladesh, respectively, compared to a diesel-only system, while the cost of electricity is reduced.

The examination of this research indicates the significance of a standalone HES configuration by including the technical, financial, and environmental effects of the proposed HES system by integrating solar PV, wind, and battery into the existing diesel generator under current conditions. This study proposes and finds the most suitable HES option for providing an economically viable, highly reliable utilizing energy from renewable resources such as wind and solar energy, while reduce the greenhouse gases emission. The research established a framework to guide the execution of the proposed HES, as well as challenges and opportunities. This study's feasibilities evaluation can be taken as a guideline for designing and implementing an off-grid application in remote locations where the utility’s grid is unavailable. Future study is needed to investigate the impact of battery charging/discharging cycles on battery lifespan and energy cost. Moreover, it is suggested that the uncertainty and prediction of power’s load should be evaluated, as well as the effect on the HES design outcomes. Finally, it could be beneficial to use more modern MCDM techniques to improve the accuracy of models.

## CRediT authorship contribution statement

**Mengly Prum:** Writing – original draft, Data curation. **Hui Hwang Goh:** Writing – review & editing, Investigation. **Dongdong Zhang:** Formal analysis. **Wei Dai:** Formal analysis. **Tonni Agustiono Kurniawan:** Conceptualization. **Kai Chen Goh:** Validation, Conceptualization.

## Declaration of competing interest

The authors affirm that they have no known financial or interpersonal conflicts that would have seemed to have an impact on the research presented in this paper.
